# Experimental Investigation of Synergistic Enhanced Oil Recovery by Infill Well Pattern and Chemical Flooding After Polymer Flooding

**DOI:** 10.3390/gels11080660

**Published:** 2025-08-19

**Authors:** Xianmin Zhang, Junzhi Yu, Lijie Liu, Xilei Liu, Xuan Lu, Qihong Feng

**Affiliations:** 1 State Key Laboratory of Deep Oil and Gas, China University of Petroleum (East China), Qingdao 266580, China; yujunzhi1031@126.com; 2 School of Petroleum Engineering, China University of Petroleum (East China), Qingdao 266580, China; 3 Research Institute of Exploration and Development, Shengli Oilfield Company, China Petroleum & Chemical Corporation, Dongying 257015, China; liulijie990.slyt@sinopec.com (L.L.); sc_lxl@163.com (X.L.); luxuan.slyt@sinopec.com (X.L.)

**Keywords:** ultra-high water cut, streamline adjustment, heterogeneous composite flooding, branched preformed particle gel, 2D visual displacement experiment, displacement front

## Abstract

Well pattern infill adjustment combined with chemical flooding is an important technical approach for significantly improving oil recovery in high-water-cut reservoirs after polymer flooding. Current research predominantly focuses on the evaluation of oil displacement potential through either well pattern infilling or chemical flooding alone, while systematic experimental investigations and mechanism studies on the synergistic effect of well pattern infilling and chemical flooding remain insufficient. To overcome the limitations of single adjustment measures, this study proposes a synergistic improved oil recovery (IOR) strategy integrating branched preformed particle gel (B-PPG) heterogeneous phase composite flooding (HPCF) with well pattern infill adjustment. Two-dimensional visual physical simulation experiments are conducted to evaluate the synergistic oil displacement effects of different displacement systems and well pattern adjustment strategies after polymer flooding and to elucidate the synergistic IOR mechanisms under the coupling of dense well patterns and chemical flooding. The experimental results demonstrate that, under well pattern infill conditions, the HPCF system exhibits significant water control and oil enhancement effects during the chemical flooding stage, achieving a 29.95% increase in stage recovery compared to the water flooding stage. The system effectively blocks high-permeability channels while enhancing displacement in low-permeability zones through a coupling effect, thereby significantly expanding the displacement sweep volume, improving displacement uniformity, and efficiently mobilizing the remaining oil in low-permeability and residual oil-rich areas. Meanwhile, well pattern infill adjustment optimizes the injection–production well pattern layout, shortens the inter-well spacing, and effectively increases the displacement pressure differential between injection and production wells. This induces disturbances and reconfiguration of the streamline field, disrupts the original high-permeability channel-dominated flow regime, further expands the sweep range of the remaining oil, and substantially improves overall oil recovery. The findings of this study enrich and advance the theoretical framework of water control and potential tapping, as well as synergistic IOR mechanisms, in high-water-cut and strongly heterogeneous reservoirs, providing a reliable theoretical and technical basis for the efficient development and remaining oil recovery in such reservoirs during the late production stage.

## 1. Introduction

During oilfield development, primary recovery that relies on natural reservoir energy and secondary recovery through water or gas injection gradually lose their effectiveness in sustaining production capacity, thereby making it increasingly difficult to meet the long-term stable production requirements of mature reservoirs [1]. As a key tertiary recovery technique, chemical flooding has been widely applied in major oilfields worldwide due to its ability to reduce oil–water interfacial tension, increase displacement fluid viscosity, and improve sweep efficiency. These effects significantly enhance the mobilization of the remaining oil, making chemical flooding essential for extending stable production periods and improving recovery in high-water-cut reservoirs [2,3,4].

Among the various chemical flooding methods, polymer flooding is the most widely applied due to its operational simplicity, strong adaptability, and favorable economics. It has been successfully implemented in major oilfields such as Shengli and Daqing in China, achieving significant incremental oil production [5,6,7]. However, influenced by reservoir heterogeneity, displacement system characteristics, well pattern configurations, and pressure distributions, substantial remaining oil bypass zones and high residual oil enrichment areas persist, even after polymer flooding. These lead to locally low displacement efficiency, severely limiting stable production and further recovery potential in late development stages [8,9].

With the progression of polymer flooding into the high-water-cut stage, flow channel solidification between injection and production wells and aggravated water channeling become increasingly evident. Consequently, a considerable amount of remaining oil is trapped in stagnant flow zones, low-permeability layers, interlayers, and unswept regions, greatly increasing the difficulty of further displacement and hindering recovery improvement [10,11,12,13]. To address the insufficient mobilization of the remaining oil after polymer flooding, extensive studies have investigated its formation mechanisms, spatial distribution, and key controlling factors. The remaining oil is generally recognized to be trapped in pore-scale residuals, blocked interlayers, dominant high-permeability flow channels, and wettability-controlled zones, exhibiting complex spatial patterns characterized by dispersion, aggregation, and multi-scale coupling behaviors [14,15,16]. Moreover, factors such as the interfacial tension, mobility ratio, well pattern, reservoir heterogeneity, and pressure system collectively influence the increasingly complex spatial distribution and enrichment of the remaining oil after polymer flooding [17,18,19]. These distributions display significant planar dispersion, interlayer variability, and aggravated intralayer heterogeneity, posing substantial challenges to reservoir management, recovery planning, and incremental recovery design.

Significant progress has been made in optimizing well patterns, diversifying displacement systems, and developing intelligent dynamic optimization methods, forming a comprehensive theoretical and technical framework for enhancing recovery after polymer flooding. These advancements have yielded promising results in multiple field applications [20,21,22]. Research shows that a significant amount of remaining oil is enriched in medium-to-low-permeability reservoirs, making them critical targets for enhanced oil recovery (EOR). To address this, various chemical flooding systems such as polymer–surfactant (SP) and alkaline–surfactant–polymer (ASP) formulations have been developed [23,24,25]. These systems effectively reduce interfacial tension and improve the mobility ratio, thereby mitigating water channeling and enhancing sweep efficiency [26]. To further optimize profile control and sweep efficiency, particulate gel systems like preformed particle gels (PPGs) have been introduced, forming heterogeneous composite flooding approaches [27,28,29]. In particular, the synergistic interaction between branched preformed particle gels (B-PPGs) and hydrolyzed polyacrylamide (HPAM) has been shown to significantly improve remaining oil mobilization after polymer flooding. Well pattern refinement and optimization strategies—including infill drilling, well spacing adjustment, and producer–injector conversion—have been employed to improve well pattern control, optimize the subsurface flow distribution, expand the sweep volume, and enhance remaining oil recovery and reservoir performance [30,31,32,33,34]. Although these measures partially improve reservoir utilization after polymer flooding, recovery enhancement is limited in the high-water-cut stage due to intensified heterogeneity, the ongoing development of high-permeability channels, and flow field solidification. To address this, Gao et al. [35] proposed a synergistic method combining profile control and plugging removal, which effectively regulates high-permeability channels, improves fluid absorption profiles, and achieved about a 12% recovery increase in laboratory tests while significantly reducing inefficient liquid production.

Intelligent dynamic optimization, particularly well placement optimization, has gained increasing attention in reservoir development to tackle dispersed remaining oil and fixed flow patterns in high-water-cut reservoirs. Various advanced algorithms have been proposed to improve both well placement strategies and displacement efficiency. Chen et al. [36] enhanced the efficiency of well placement optimization in heterogeneous reservoirs by integrating an analytical objective function with the Cat Swarm Optimization (CSO) algorithm. Naderi [37] employed the Bat algorithm for well placement optimization, demonstrating its ability to preserve the global search capability while achieving a higher net present value (NPV) uplift than the conventional genetic algorithm (GA) and particle swarm optimization (PSO) methods. With an increasing optimization complexity, multi-objective algorithms like NSGA-III have been employed to dynamically balance key development indicators, including the recovery factor, production rate, and water cut [38]. To further reduce computational costs and improve efficiency, machine learning-assisted proxy models have been progressively incorporated into optimization workflows. For example, Davudov et al. [39] developed a Fast Marching Method (FMM)-based proxy model, significantly improving both optimization speed and predictive accuracy. More recently, deep learning and transfer learning frameworks have also been introduced to well placement and reservoir development optimization, particularly under complex and highly heterogeneous geological conditions.

Meanwhile, in the context of chemically flooded reservoirs during the late development stages, infill well pattern chemical flooding has emerged as an important technique for enhancing oil recovery. By optimizing well density, improving injection–production relationships, and expanding the chemical sweep volume, this approach has proven effective in mobilizing residual oil, particularly in interlayered and low-permeability zones, thereby promoting more balanced reservoir development [40,41,42,43]. In an early field application, Gu et al. [44] implemented a small well spacing five-spot pattern combined with ASP ternary flooding in Karamay Oilfield, achieving a 15% increase in oil recovery and verifying its technical and economic feasibility in heterogeneous conglomerate reservoirs. Similarly, Zhang et al. [45] conducted an ASP flooding pilot in a high-water-cut block of Daqing Oilfield, where the use of blended biosurfactants alongside conventional surfactants achieved ultra-low interfacial tension while reducing chemical costs and injection pressures, and it resulted in a 16.64% improvement in oil recovery and a 35.5% reduction in the water cut of central wells. Furthermore, Onwunalu et al. [46] analyzed production data and numerical simulations of a polymer-flooded reservoir in Argentina, and they found that reducing the well spacing can significantly improve sweep efficiency, confirming the importance of infill well pattern adjustment for mitigating bypassed oil in the later stages of polymer flooding. In another field case, Sun et al. [47] applied a combined strategy of well pattern adjustment and heterogeneous composite flooding in Gudao Oilfield, which increased oil recovery by 3.5%, reduced the water cut by 18.5%, and raised daily oil production by 81.2 t.

A substantial number of laboratory investigations have further validated the feasibility of integrating chemical flooding with well pattern adjustment while deepening the understanding of its displacement mechanisms. Li et al. [48] demonstrated via a two-dimensional visual model that polymer flooding alone exerts limited control over flow field configurations, whereas well pattern adjustment can effectively restructure streamlines and mobilize residual oil in off-mainstream regions, highlighting its necessity during ultra-high-water-cut stages. Building upon this, Tao et al. [49] combined laboratory experiments with numerical simulations to reveal that integrating SP composite flooding with infill well pattern adjustment and streamline reorientation significantly improves sweep efficiency in highly heterogeneous reservoirs, with residual oil saturation quantitatively measured using an electrical resistance probe. Further extending the concept, Jiao et al. [50] proposed a streamline transformation–well pattern matching strategy based on a three-dimensional heterogeneous physical model, achieving enhanced coupling between displacement directions and residual oil-enriched zones, thereby substantially expanding the effective sweep volume. Du et al. [51] investigated the synergistic effects of B-PPG, polymer, and surfactant ternary flooding under optimized well patterns, while Zhang et al. [52] introduced a heterogeneous composite system that redirected dominant streamlines, effectively plugging high-permeability channels and enhancing displacement in stagnant zones. He et al. [53] conducted large-scale sand-packed experiments to compare the performance of well pattern adjustment (WPA), heterogeneous composite flooding (HPCF), and their combined application, confirming that the integrated scheme jointly mobilized residual oil in both dominant and off-dominant streamline regions, delivering superior oil recovery over single techniques. However, the spatial distribution of the remaining oil after polymer flooding remains highly complex, particularly under high-water-cut conditions and pronounced reservoir heterogeneity. This distribution is jointly controlled by multiple interacting factors, including the well pattern configuration, chemical flooding system performance, displacement pressure field, and reservoir heterogeneity. Consequently, infill chemical flooding processes exhibit a significant spatial migration of the remaining oil, dynamic enrichment, and marked variations in displacement efficiency. Currently, systematic experimental investigations into the macroscopic evolution and synergistic mobilization mechanisms of the remaining oil during infill chemical flooding are still lacking. In particular, comprehensive studies on the mobilization pathways and dynamic evolution patterns of the remaining oil under the coupled influence of multiple factors remain insufficient, which has become a key bottleneck limiting the continuous enhancement of infill chemical flooding performance.

Therefore, this study focuses on elucidating the synergistic displacement mechanisms of infill well pattern adjustment combined with heterogeneous composite flooding. Through large-scale two-dimensional visual physical simulation experiments, the displacement characteristics under various combinations of displacement agents and infill well pattern configurations are systematically compared. The spatial distribution patterns of residual oil enrichment zones are characterized, and the mechanisms by which the synergistic technique enhances sweep efficiency, improves residual oil mobilization, and reconstructs streamline configurations in post-polymer flooding reservoirs are further clarified. The outcomes of this study provide important theoretical support and engineering guidance for optimizing infill well pattern designs, formulating effective residual oil tapping strategies, prolonging the stable production period of mature oilfields, and improving overall reservoir recovery efficiency.

## 2. Results and Discussion

### 2.1. Comparative Rheological Analysis of Displacement Agents

The rheological behavior of displacement agents plays a pivotal role in determining oil recovery efficiency, as their dynamic viscoelastic properties critically influence the transport behavior and sweep efficiency of the displacing fluid within porous reservoir media. To systematically evaluate the adaptability and displacement potential of various flooding systems under reservoir conditions, this study conducted a comprehensive rheological characterization, including steady-shear measurements, dynamic oscillatory tests, and long-term stability analyses. The results revealed pronounced differences among the systems in terms of the structural stability, viscoelastic response, and interfacial modulation capacity. These findings provide essential theoretical support and technical guidance for the selection and application of high-efficiency flooding agents in reservoirs following polymer flooding.

#### 2.1.1. Steady-Shear Rheology of the Three Displacement Systems

**Figure 1:**
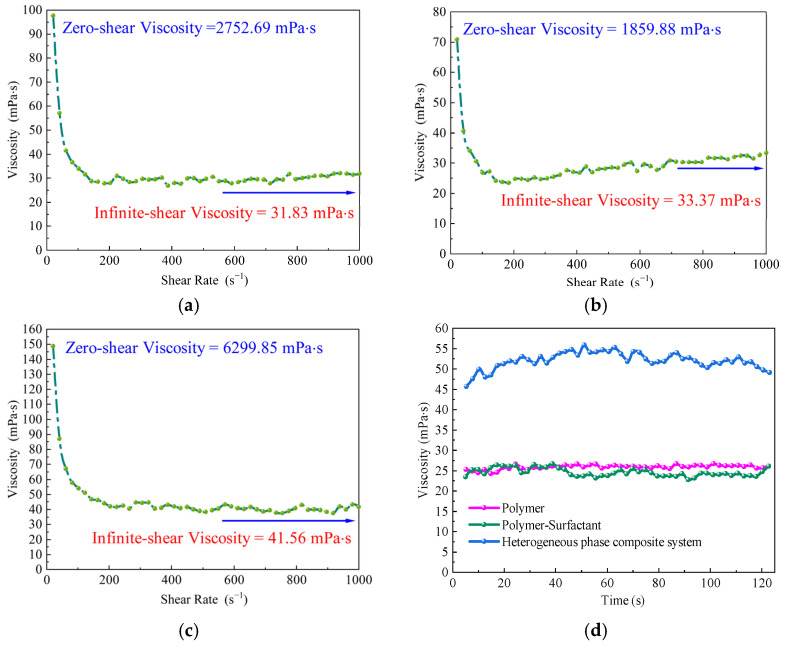
Steady-shear rheological behavior of the three displacement systems. (**a**) Viscosity variation of the polymer system under different shear rates. (**b**) Viscosity variation of the binary composite system under different shear rates. (**c**) Viscosity variation of the heterogeneous composite system under different shear rates. (**d**) Viscosity evolution of the three systems under constant-shear conditions over time.

All three displacement systems exhibit typical shear-thinning behavior, as evidenced by a significant reduction in viscosity with an increasing shear rate (Figure 1). This rheological characteristic can directly impair the displacement efficiency of the injectant within the reservoir. Effective oil displacement relies on the fluid maintaining sufficient viscosity in porous media to establish a stable pressure gradient capable of driving crude oil toward production wells. The zero-shear viscosities of the polymer and binary composite systems are 2752.69 mPa·s and 1859.88 mPa·s, respectively; however, both rapidly decline to approximately 30 mPa·s under high-shear conditions, indicating that their internal structures are susceptible to shear-induced degradation and thus cannot maintain an effective displacement capability in heterogeneous porous formations.

In contrast, the heterogeneous composite system demonstrates a significantly higher zero-shear viscosity of 6299.85 mPa·s and sustains elevated viscosity across the full shear rate spectrum (Table 1). Under constant-shear conditions, its viscosity stabilizes at 52.03 mPa·s—substantially higher than that of the other two systems. These findings suggest that the heterogeneous system features a more robust and interconnected three-dimensional network, conferring enhanced shear resistance. This enables the fluid to maintain a continuous pressure gradient throughout the displacement process, stabilize the displacement front, enlarge the sweep volume, and suppress viscous fingering. As a result, it represents a more effective strategy for mobilizing dispersed residual oil in post-polymer flooding reservoirs.

**Figure 2:**
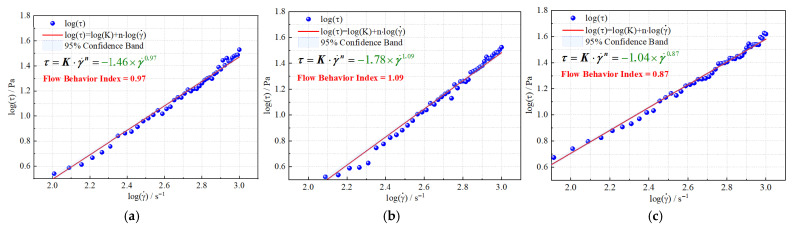
Comparison of flow behavior index fitting curves for the three displacement systems. (**a**) Flow behavior index fitting curve of the polymer displacement system. (**b**) Flow behavior index fitting curve of the binary composite displacement system. (**c**) Flow behavior index fitting curve of the heterogeneous composite displacement system.

Moreover, the heterogeneous composite system exhibits a flow behavior index (n) of only 0.87, which is significantly lower than that of the polymer (0.97) and binary composite systems (1.09), indicating a slower rate of viscosity decline under increasing shear and greater structural stability (Figure 2). This low n value reflects excellent shear stability and structural retention. Typically, such rheological characteristics suggest the formation of a denser and more highly cross-linked three-dimensional network within the fluid, which can effectively resist shear-induced structural breakdown. As a result, the system can maintain its viscoelastic support, enhance the stability of the displacement front, and improve sweep efficiency during the oil displacement process [54,55].

#### 2.1.2. Shear Hysteresis Characteristics of the Three Displacement Systems

**Figure 3:**
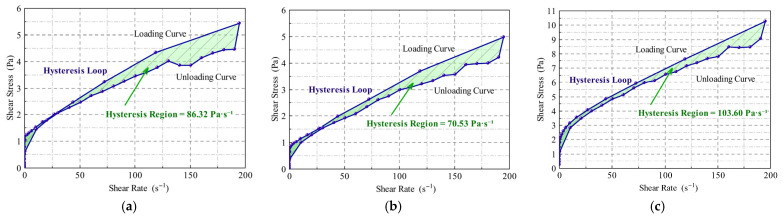
Shear hysteresis behavior of the three oil displacement systems. (**a**) Shear hysteresis loop of the polymer solution system. (**b**) Shear hysteresis loop of the binary composite flooding system. (**c**) Shear hysteresis loop of the heterogeneous composite flooding system.

A comparative analysis of the rheological differences among the three displacement systems was conducted using shear hysteresis loop tests (Figure 3). The results show that the heterogeneous composite system exhibits the most favorable overall performance. Although the differences in the hysteresis loop area among the systems are relatively small, the heterogeneous system displays a slightly larger area. Combined with its structural recovery rate exceeding 87% and high viscosity levels, this system demonstrates a distinctive “high dissipation-rapid recovery” characteristic. This indicates its strong capacity for energy dissipation during shear deformation and the rapid reformation of its internal network structure upon unloading, reflecting superior shear responsiveness and viscoelastic adaptability. During the oil displacement process, such behavior offers dual advantages: on the one hand, high viscosity facilitates the establishment of a sustained and effective pressure gradient, thereby enhancing displacement efficiency; on the other hand, robust structural recovery contributes to the morphological stability of the displacement front, improving sweep efficiency and promoting the mobilization of residual oil.

#### 2.1.3. Dynamic Viscoelastic Behavior of the Three Displacement Systems

**Figure 4:**
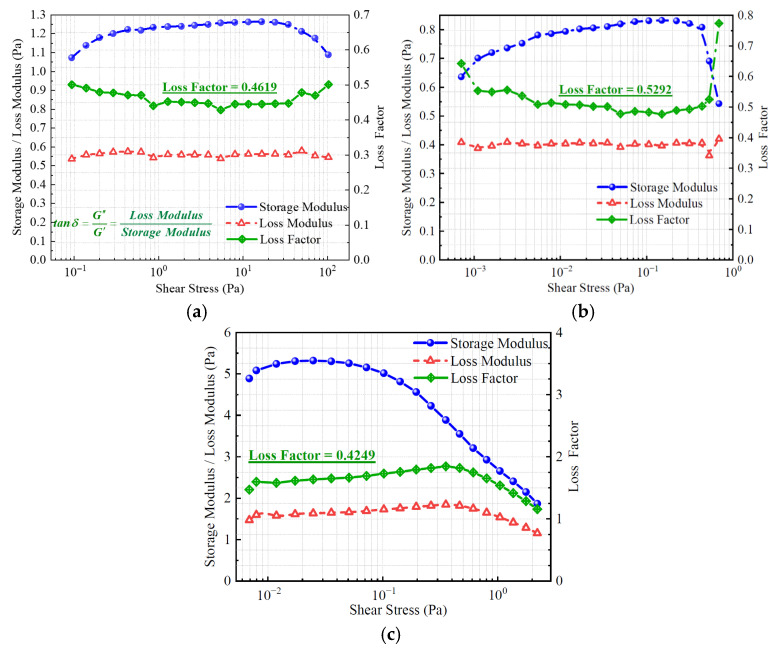
Dynamic viscoelastic parameter analysis of the three displacement systems. (**a**) Variations in the storage modulus, loss modulus, and loss factor of the polymer solution system. (**b**) Variations in the storage modulus, loss modulus, and loss factor of the binary composite displacement system. (**c**) Variations in the storage modulus, loss modulus, and loss factor of the heterogeneous composite displacement system.

Figure 4 illustrates the variations in the storage modulus, loss modulus, and loss factor of the three displacement systems under different shear stress conditions. All systems exhibit a storage modulus greater than the loss modulus, indicating that elastic behavior dominates and that the systems possess strong structural support and shape retention capabilities. Among them, the heterogeneous composite system consistently shows the highest storage modulus across the entire stress range, reflecting a denser internal network structure, superior energy storage capacity, and higher structural rigidity. In contrast, the polymer and binary composite systems display lower storage moduli, suggesting limited elastic contributions and weaker structural stability. In terms of the loss factor, the binary composite system exhibits the highest average value (approximately 0.5292), followed by the polymer system (0.4619) and the heterogeneous composite system (0.4249). A higher loss factor generally reflects greater energy dissipation and more pronounced viscous behavior. For oil displacement applications, an appropriate level of viscosity is beneficial for improving the compatibility between the displacing fluid and porous media. However, an excessively high loss factor may compromise structural integrity and weaken the displacement strength.

Overall, the heterogeneous composite displacement system exhibits the most favorable dynamic viscoelastic performance. It demonstrates significantly higher storage and loss moduli than the other systems while maintaining a moderate loss factor. This indicates a well-balanced interplay between elastic and viscous responses. Such rheological characteristics suggest that the system can efficiently store mechanical energy and maintain structural stability under shear disturbances. These advantages make it particularly suitable for post-polymer flooding applications under complex reservoir conditions, enhancing its adaptability and displacement efficiency in heterogeneous formations [56,57].

#### 2.1.4. Surface and Interfacial Tension Regulation of the Three Displacement Systems

**Figure 5:**
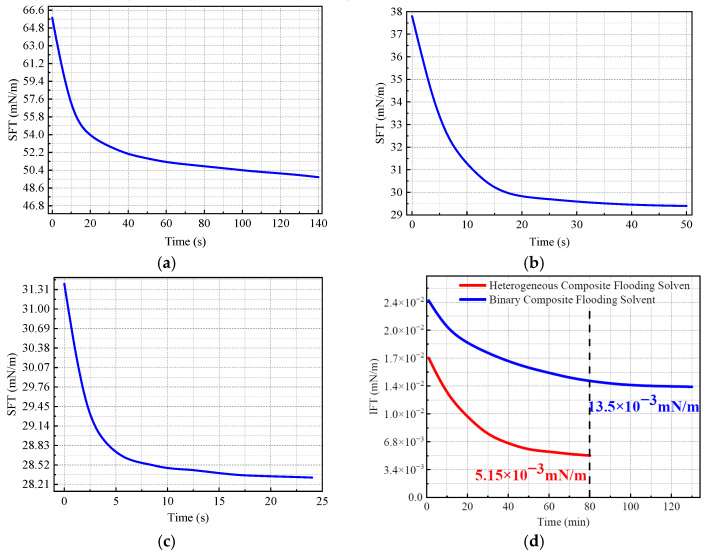
Temporal evolution of the surface and interfacial tension of the three displacement systems. (**a**) Surface tension variation of the polymer solution system. (**b**) Surface tension variation of the binary composite system. (**c**) Surface tension variation of the heterogeneous composite system. (**d**) Interfacial tension variation between the binary and heterogeneous composite systems over time.

The experimental results demonstrate that all three displacement systems exhibit some effectiveness in reducing surface and interfacial tensions; however, the heterogeneous composite system shows superior performance. In terms of surface tension, this system exhibits the lowest initial value and reaches equilibrium rapidly, indicating excellent surfactant diffusion kinetics and interfacial saturation behavior. Regarding interfacial tension, its final value drops to as low as 5.15 × 10^−3^ mN/m, which is significantly lower than that of the binary composite system (13.5 × 10^−3^ mN/m) (Figure 5). This reduction in interfacial energy effectively facilitates the emulsification and detachment of residual oil, thereby enhancing oil displacement efficiency.

Overall, the three systems exhibit notable differences in rheological behavior, dynamic viscoelasticity, and interfacial regulation performance, with the heterogeneous composite system consistently outperforming the others. It possesses the highest zero-shear viscosity and the strongest shear resistance, enabling the establishment and maintenance of effective pressure gradients throughout the porous reservoir. Additionally, its “high energy dissipation–rapid recovery” rheological profile endows it with excellent structural reconstruction capabilities and enhanced displacement front stability. Dynamic oscillatory testing reveals that this system exhibits a significantly higher storage modulus than the other systems, indicating superior structural rigidity and elasticity. Moreover, the heterogeneous system rapidly reduces both surface and interfacial tensions, improving oil–water interfacial interactions and further enhancing displacement efficiency. Taken together, these findings suggest that the heterogeneous composite system offers greater adaptability and displacement potential under complex reservoir conditions, making it a promising candidate for enhanced oil recovery following polymer flooding [58,59,60].

### 2.2. Infill Well Pattern Flooding Experiments with Various Chemical Systems

**Figure 6:**
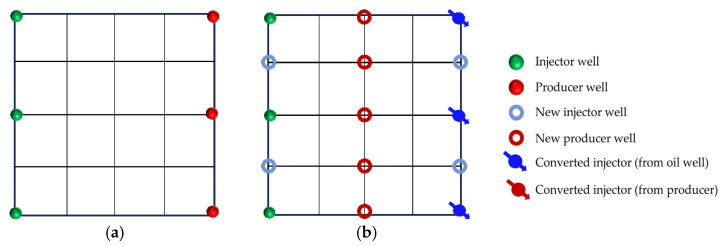
Changes in well pattern configuration before and after infill drilling. (**a**) Original line-drive pattern with a spacing of 300 × 150 m. (**b**) Line-drive pattern after infill adjustment with a spacing of 150 × 75 m.

In reservoirs with well-developed high-permeability streaks, long-term water injection aggravates reservoir heterogeneity and causes significant variations in areal sweep efficiency. This study aims to investigate the influence of injecting different displacement agents and the timing of well infill on the synergistic performance of chemical flooding combined with dense well pattern technology. The research focuses on the synergistic improvement effects of various displacement strategies in terms of regulating dominant flow channels, mobilizing residual oil in low-permeability zones, and enhancing overall displacement efficiency so as to clarify the applicability and displacement mechanisms of “chemical flooding + well pattern infill” synergistic technology. The objective is to provide a theoretical basis and technical support for the efficient development of heterogeneous reservoirs during the high-water-cut stage. Based on the actual well pattern and production system of a certain block in Shengli Oilfield, the original well pattern is designed as a line-drive pattern with a spacing of 300 × 150 m. After infill drilling, it remains a line-drive well pattern but with a reduced spacing of 150 × 75 m (as shown in Figure 6). According to the types of displacement agents and the timing of infill drilling implementation, four sets of comparative experiments are designed:

Scheme 1: Water flooding followed by well pattern infill for binary composite flooding (WF-WAPSF);

Scheme 2: Water flooding followed by well pattern infill for heterogeneous composite flooding (WF-WAHPCF);

Scheme 3: Polymer flooding after water flooding, followed by well pattern infill for heterogeneous composite flooding (WF-PF-WAHPCF);

Scheme 4: Binary composite flooding after water flooding, followed by well pattern infill for heterogeneous composite flooding (WF-PSF-WAHPCF).

**Figure 7:**
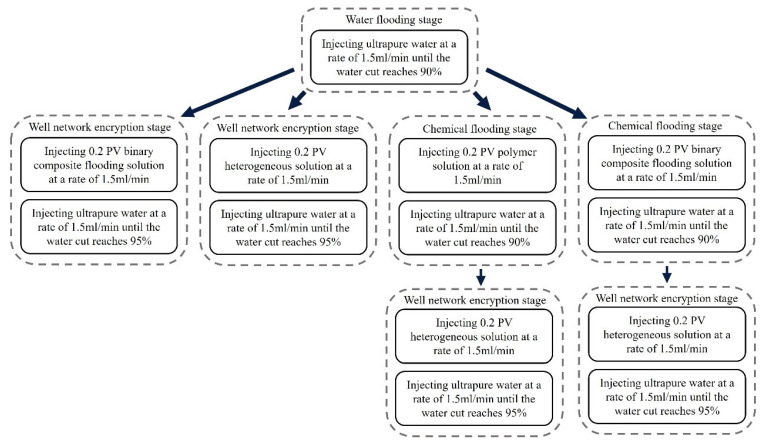
Schematic diagram of displacement procedures in the experimental design.

The experimental procedures for the different displacement systems are illustrated in Figure 7.

#### 2.2.1. Visual Results During the Water Flooding Stage

**Figure 8:**
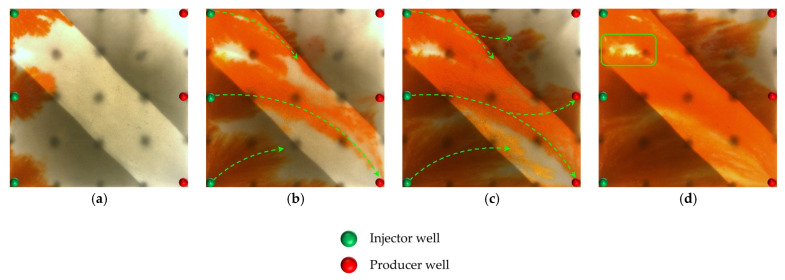
Dynamic evolution of water flooding in a reservoir model with developed high-permeability streaks. (**a**) Oil–water distribution at 0.1 pore volume (PV) injection. (**b**) Oil–water distribution at 0.3 PV injection. (**c**) Sweep status at 0.6 PV injection. (**d**) Residual oil distribution at 0.8 PV injection.

In the heterogeneous model with developed high-permeability streaks, due to the significant viscosity contrast between oil and water (oil viscosity: 45 mPa·s; water viscosity: 0.5 mPa·s), the waterflood front exhibits a typical viscous fingering pattern during the displacement process, characterized by a pronounced non-uniform advancement. As shown in Figure 8, because of the good pore connectivity in the high-permeability streaks, the injected water preferentially displaces along these paths, resulting in early water breakthrough in the production wells located in these regions. In contrast, the production wells in low-permeability zones experience delayed water breakthrough, leading to a limited sweep efficiency. Consequently, the overall water cut rapidly increases to 28.6% (as shown in Figure 8b). After water breakthrough, a dominant seepage channel is first formed within the high-permeability streaks. Under the combined influence of pressure interference between the injection and production wells and reservoir heterogeneity, the structure of the reservoir pressure field changes, resulting in the deflection of the water injection streamlines. The diversion lines extend outward along the main flow channel, leading to a 30.05% expansion in the sweep area (Table 2). Under long-term water flooding, with the advancement of injection and the intensification of channeling, the flow path gradually evolves into a high-water-consuming zone, causing inefficient water circulation and a significant decline in oil displacement efficiency, thereby exacerbating the water channeling problem in the later stage. As a result, a large amount of unswept residual oil remains in low-permeability zones, becoming a key factor restricting the further improvement of recovery efficiency.

At the end of water flooding, the stage recovery factor ultimately stabilizes at 32.32%. The remaining oil is mainly distributed along the model boundaries, between the injection wells, and around the production wells located in low-permeability zones. These areas commonly exhibit regions not swept by the injected water, and they macroscopically show a pattern of continuous and concentrated residual oil accumulation, indicating low sweep efficiency at this stage. In addition, in locally weakly swept zones within the high-permeability streaks, the remaining oil is primarily retained within the pore–throat structures, further limiting the improvement of displacement efficiency.

**Figure 9:**
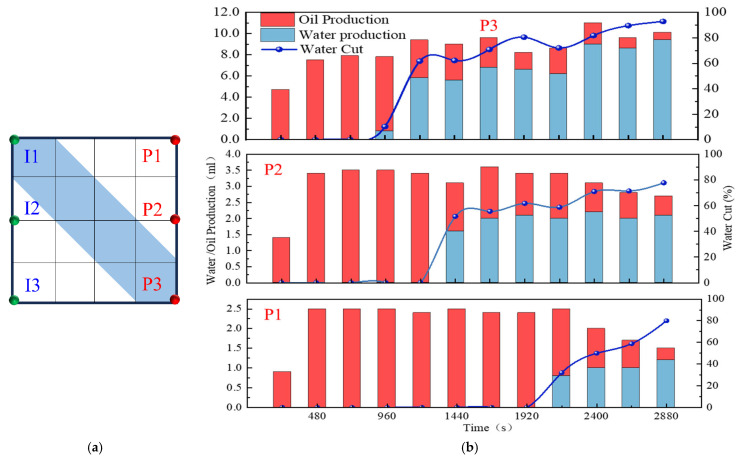
Single-well production performance curves during the water flooding stage in the reservoir model with developed high-permeability streaks. (**a**) Well placement. (**b**) Variations in single-well oil production, water production, total liquid production, and water cut.

Based on the oil production, liquid production, and water cut variation curves shown in Figure 9b, the model is affected by high-permeability streaks, and the production dynamics of individual wells exhibit significant heterogeneity. Well P3, as the main production well, accounts for 62.5% of the total liquid production in the model, which is significantly higher than the combined liquid production of wells P1 and P2. During the water injection development process, the water-free oil production period extends to an injection volume of 0.3 PV. After water breakthrough, the water cut of wells P1 and P2 rapidly increases to 60% within 0.4 PV, reflecting the preferential breakthrough of high-permeability channels, while well P3, due to delayed displacement in the low-permeability zone, shows a slowly increasing trend in the water cut. In terms of oil recovery efficiency, the main oil production period is concentrated in the medium-to-low-water-cut stage (water cut < 60%), contributing nearly 70% of the model’s cumulative oil production, whereas in the high-water-cut stage (>60%), oil production declines significantly.

**Figure 10:**
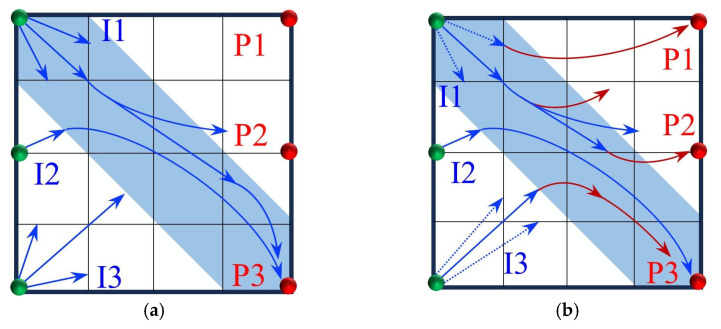
Streamline evolution during the water flooding stage in the reservoir model with developed high-permeability streaks. (**a**) Distribution of main streamlines in the early stage of water injection. (**b**) Expansion of main streamlines in the late stage of water injection.

The streamline evolution during the water flooding stage is shown in Figure 10. In the early stage of water injection (0–0.3 PV), the injected water is primarily distributed along high-permeability channels. The streamline density in the low-permeability zone around well P1 is lower than that in the high-permeability region, and the model exhibits a distinct non-uniform distribution. As water injection progresses (0.3–0.8 PV), the sweep area of the streamlines expands, with the streamlines mainly concentrated within the high-permeability channels. In the late stage of water injection, the oil production rate of wells P1 and P2 decreases, while that of well P3 increases. A dominant seepage channel gradually forms, leading to flow convergence and inefficient circulation of the injected water.

#### 2.2.2. Visual Results During the Post-Waterflood Infill Chemical Flooding Stage

**Figure 11:**
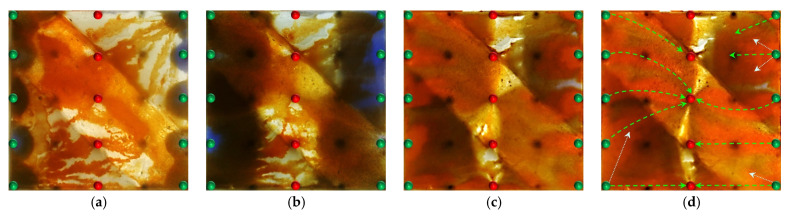
Displacement process during the WAPS stage (WF–WAPSF). (**a**) Initial stage of PS injection. (**b**) Final stage of PS injection. (**c**) Initial stage of subsequent water injection. (**d**) The 95% water cut stage.

**Figure 12:**
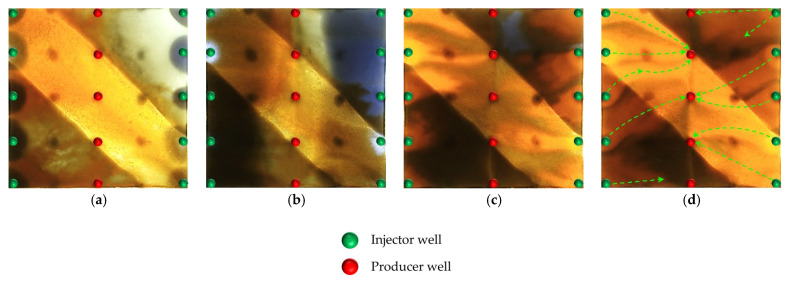
Displacement process during the WAHPC stage (WF–WAHPCF). (**a**) Initial stage of HPC injection. (**b**) Final stage of HPC injection. (**c**) Initial stage of subsequent water injection. (**d**) The 95% water cut stage.

Figure 11 and Figure 12 illustrate the macroscopic displacement effects of implementing infill well patterns combined with binary compound flooding or heterogeneous flooding after water flooding. As shown in Figure 11d and Figure 12d, the non-uniform sweep phenomenon observed after water flooding is significantly mitigated following the implementation of chemical flooding with infill well patterns. After well pattern adjustment, the original production wells are converted into injection wells, and the injection–production streamlines are redirected, effectively mobilizing residual oil in the near-well regions toward the production wells. Meanwhile, reducing the well spacing enhances the control capability of individual wells, and increasing the number of wells weakens the interference between the injection wells. Chemical flooding increases the viscosity of the aqueous phase and reduces its mobility, thereby improving the oil–water mobility ratio, mitigating the fingering phenomenon, increasing sweep efficiency, and enhancing the overall displacement efficiency (Table 3).

Compared with binary compound flooding, heterogeneous flooding exhibits significant advantages due to the incorporation of a PPG (pre-crosslinked particle gel). The PPG selectively plugs high-permeability channels and reconstructs the displacement flow field. In synergy with the surfactant/polymer, it forms a “plug–adjust–displace” system, which builds a three-dimensional mesh structure within the reservoir to alter the flow paths of fluids. Through swelling and interconnection, the PPG effectively blocks high-permeability streaks, thereby reducing the non-selective flow of the aqueous phase. This not only mobilizes unswept residual oil in uninvaded zones and improves the sweep efficiency of the model but also displaces the discontinuous residual oil remaining in strongly water-washed zones, enhancing the overall displacement efficiency and ultimately resulting in a significant increase in the stage recovery factor. Although heterogeneous compound flooding can achieve remarkable results through the use of the PPG, in the subsequent water flooding stage, the injected water first breaks through the chemical flooding slug in the high-permeability zone. During the ultra-high-water-cut stage, the slug remains in low-permeability regions and between the main and diverted flow lines, further increasing the seepage resistance in low-permeability zones. This aggravates reservoir heterogeneity and leads to stagnation in recovery factor improvement.

In high-permeability channels, the binary compound flooding system is limited by an insufficient mobility control capability, resulting in the injected water breaking through the chemical slug and causing a distinct slug retention phenomenon between the diversion lines. In contrast, the heterogeneous compound flooding system, by constructing a higher effective viscosity and a three-dimensional network structure, enables more complete displacement of the chemical slug by the injected water. In low-permeability zones, the plugging capability of the heterogeneous system is stronger than that of the binary system, requiring higher displacement pressure to mobilize the slug. Due to increased slug retention, reservoir heterogeneity is further aggravated.

#### 2.2.3. Visual Results of the Post-Waterflood Chemical Flooding Stage

**Figure 13:**
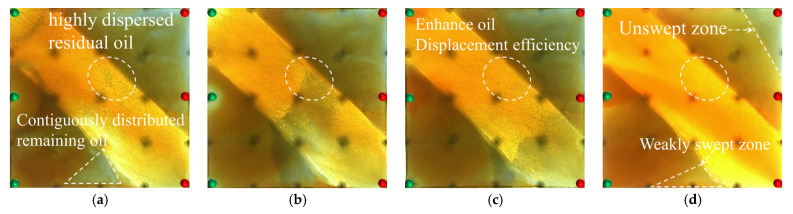
Displacement process during the PF stage (WF–PF–WAHPCF). (**a**) Initial stage of polymer injection. (**b**) Final stage of polymer injection. (**c**) Initial stage of subsequent water injection. (**d**) The 95% water cut stage.

**Figure 14:**
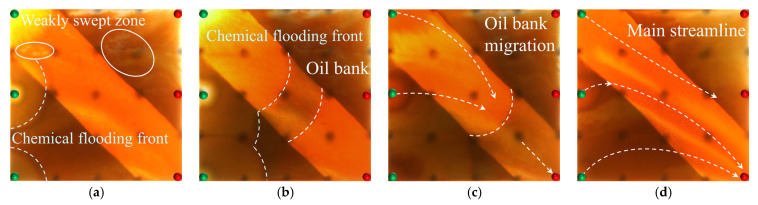
Displacement process during the PS stage (WF–PSF–WAHPCF). (**a**) Initial stage of PS injection. (**b**) Final stage of PS injection. (**c**) Initial stage of subsequent water injection. (**d**) The 95% water cut stage.

As shown in Figure 13 and Figure 14, polymer flooding and binary compound flooding were conducted based on prior water flooding. The results indicate that the two displacement methods exhibited significant differences in macroscopic sweep behavior and residual oil distribution characteristics. At the initial stage of chemical slug injection, the increased system viscosity effectively optimized the oil–water mobility ratio and improved the advancing morphology of the displacement front. At this stage, the distribution of flow resistance was relatively uniform, and the front of the slug expanded in a regular fan-shaped pattern, effectively suppressing the fingering phenomenon of the injected water within high-permeability channels. This promoted the migration of residual oil toward the production wells, thereby significantly enhancing the displacement efficiency within the high-permeability channels. Meanwhile, residual oil accumulated at the front of the slug, forming high-oil-saturation bands. With an increasing slug injection volume, the area of these streaks gradually expanded and exhibited a clear enrichment pattern within the high-permeability channels. However, in the late stage of slug propagation (as shown in Figure 13d and Figure 14d), due to reservoir heterogeneity, the displacement capability in low-permeability zones was insufficient, leading to the formation of weakly swept regions, where residual oil accumulated in a continuous patch-like manner. This indicates that, although polymer flooding can improve displacement efficiency in the main flow channels, it still shows certain limitations in mobilizing oil in low-permeability regions. In the subsequent water flooding stage, the injected water continued to drive the chemical slug toward the production wells, effectively mobilizing the high-oil-saturation streaks at the slug front. This further improved the displacement efficiency within the high-permeability channels and, to some extent, expanded the sweep range between the injection and production wells, as well as in the low-permeability zones, achieving localized displacement compensation.

However, as displacement entered the later stage, the reduction in the chemical agent concentration and the decline in the displacement capability caused the injected water to preferentially flow along the high-permeability channels. Dominant seepage pathways were re-established, resulting in a decreased water circulation efficiency and a limited improvement in sweep efficiency. Meanwhile, since the injection–production well pattern was not further optimized, a stable distribution of the main streamlines persisted, leading to insufficient drive between the wells. The phenomena of unswept and weakly water-washed conditions remained prominent in low-permeability zones, where residual oil was retained in dispersed and bound forms, becoming a key obstacle to further improvement in the overall recovery factor.

Overall, polymer flooding performs well in enhancing the oil displacement efficiency within high-permeability streaks and suppressing the fingering of injected water, making it suitable for flow control in reservoirs with developed high-permeability channels. In contrast, binary compound flooding demonstrates superior performance in constructing a stable displacement front, enhancing oil bank migration, and improving the mobilization of low-permeability zones. However, both displacement methods face challenges in the later stages of heterogeneous reservoirs, including the re-establishment of dominant flow channels and insufficient mobilization in low-permeability regions. Therefore, further improvements in synergistic displacement efficiency and recovery potential require the optimization of heterogeneous compound flooding and adjustments to the injection–production well pattern layout.

**Figure 15:**
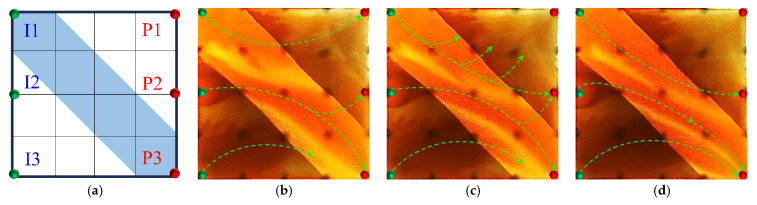
Streamline evolution during the subsequent water injection stage after chemical flooding in the reservoir model with developed high-permeability streaks. (**a**) Schematic Diagram of a Reservoir Model with Developed High-Permeability Streaks. (**b**) Early stage of subsequent water injection. (**c**) Late stage of subsequent water injection. (**d**) Formation stage of dominant seepage channels.

As shown in Figure 15, during the binary compound flooding stage, the dynamic evolution of the main streamlines exhibits distinct stage-dependent characteristics. In the early stage of subsequent water injection, due to the blocking effect of the binary compound slug in the high-permeability region, the injected water from well I1 is hindered and preferentially flows toward well P1. With an increasing injection volume, the slug gradually enters the low-permeability zone, where the flow resistance increases, causing the water flow path to shift toward the direction of lower resistance. As a result, the injection–production streamline from well I1 deviates and tends toward well P2. After the injected water breaks through the slug, a dominant seepage channel is formed, and water preferentially advances along this dominant path. Consequently, the weakly swept area around well P1 remains difficult to effectively mobilize, significantly limiting the effectiveness of the water flooding.

At the initial stage of slug blocking, the injection streamlines from well I2 simultaneously act on both wells P2 and P3, achieving a certain degree of balanced sweep. However, with changes in the pressure gradient between the injection and production wells, the injection resistance decreases, and the water flow gradually shifts toward well P3. As a result, the streamlines from well I2 bifurcate, flowing separately toward the two production wells. In contrast, well I3 exhibits different displacement characteristics. During the early stage of water injection, the injected water displaces relatively uniformly in the low-permeability zone. However, as the injection volume increases, the flow gradually enters the high-permeability region, and the main streamlines clearly shift toward well P3. Meanwhile, chemical agent retention zones gradually form between the main streamlines, reducing the local displacement efficiency and hindering the further advancement of the chemical slug.

Based on an analysis of streamline variations across the wells, it is observed that the overall displacement process exhibits a staged evolution from balanced and dispersed flow to concentrated flow along dominant channels. In the initial stage, the distribution of the injected water streamlines is relatively dispersed, with no dominant seepage channels yet formed, and the displacement front advances uniformly. As the injection volume increases, reservoir heterogeneity becomes more pronounced, causing partial streamline deflection, and high-permeability zones gradually become the dominant flow paths. In the later stage of displacement, dominant seepage channels are established, the water flow becomes more concentrated, channeling intensifies, and weak sweep in low-permeability zones becomes prominent. The residual oil remains primarily in dispersed and bound forms, leading to a certain degree of limitation on the overall displacement efficiency.

**Figure 16:**
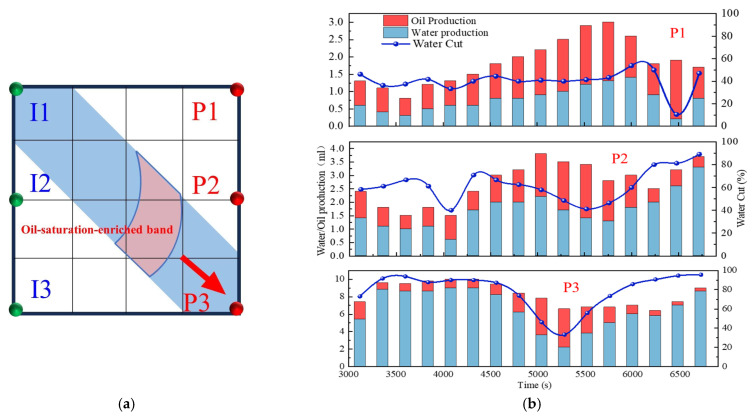
Chemical flooding process in the reservoir model with developed high-permeability streaks. (**a**) Migration of high-oil-saturation streaks along high-permeability zones. (**b**) Single-well production performance curves during the binary compound flooding stage.

According to the single-well production performance curves in Figure 16b, the three production wells exhibit significant differences during the chemical flooding stage. For well P1, the water cut initially remains stable at around 60%, and then it decreases in the later stage to a minimum of 10.92%. The liquid production rate shows a trend of first increasing and then declining, indicating that the chemical slug effectively plugged the high-permeability channels and mobilized the residual oil in the low-permeability zones. Well P2 shows the earliest reduction in the water cut, dropping from 66.5% to 40%, corresponding to the preferential production of a high-oil-saturation streak. As the chemical slug migrates, the liquid production rate fluctuates within the range of 1.5–3.6 mL/min. Well P3 exhibits a typical three-stage behavior characterized as “high-water-cut steady state, water cut funnel, and formation of a dominant flow channel.” In the initial stage, the water cut remains above 80%; during the production of the oil saturation streak, the water cut decreases to below 40%. With continued water injection, the water cut rises again, and the liquid production rate shows a trend of initial stability, followed by a decline, and then an increase in the later stage.

#### 2.2.4. Visual Results of Infill Well Pattern Heterogeneous Flooding After Chemical Flooding

Building upon the clear differences observed in the early-stage chemical flooding, a systematic analysis was further conducted on two experimental schemes during the infill heterogeneous flooding stage. Although the same heterogeneous compound slug system was used in this stage, the displacement performance still showed significant differences. Scheme 4 outperformed Scheme 3, reflecting the inherited and amplified effect of the early-stage displacement state on the supplemental drive efficiency of infill chemical flooding. The infill well pattern technology, by increasing the well density by 40% and reducing the control area per well, significantly enhanced the flow field control capability and improved the streamline distribution and connectivity, thereby creating favorable conditions for synergistic heterogeneous flooding. After the injection of the heterogeneous compound slug, it preferentially formed a dense distribution within the high-permeability streaks, effectively increasing seepage resistance in this zone, suppressing unidirectional water breakthrough, and promoting fluid diversion into low-permeability regions.

**Figure 17:**
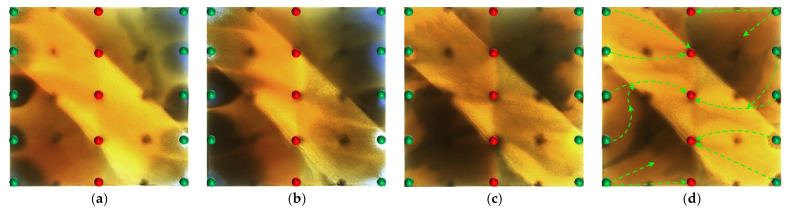
Displacement process during the WAHPC stage (WF-PF-WAHPCF). (**a**) Initial stage of HPC injection. (**b**) Final stage of HPC injection. (**c**) Initial stage of subsequent water injection. (**d**) The 95% water cut stage.

**Figure 18:**
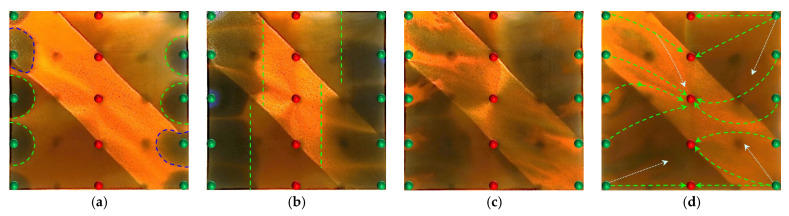
Displacement process during the WAHPC stage (WF-PSF-WAHPCF). (**a**) Initial stage of HPC injection. (**b**) Final stage of HPC injection. (**c**) Initial stage of subsequent water injection. (**d**) The 95% water cut stage.

However, the distribution pattern of the residual oil formed during the early stage by different chemical flooding systems had a significant impact on the effectiveness of the supplemental drive in the infill stage. In Scheme 3, severe fingering occurred in the early stage, with fully developed dominant channels, leading to substantial slug retention in high-permeability zones. Poor connectivity in low-permeability areas caused the streamlines to become concentrated during the subsequent water flooding stage, resulting in uneven displacement and poor mobilization of weakly swept zones (Figure 17). In contrast, Scheme 4, with its binary system, exhibited pronounced emulsification and flow diversion effects in the early stage, leading to a more stable displacement front, weaker development of dominant channels, and better mobilization in low-permeability regions (Figure 18). During the infill water flooding stage, the water flow was more evenly distributed, and the supplemental displacement performance significantly improved.

In summary, although the process and system used in the infill well pattern heterogeneous compound flooding stage were the same, the difference in displacement conditions during the early-stage chemical flooding was the fundamental reason for the difference in displacement performance. Scheme 4, relying on favorable early-stage sweep efficiency and an optimized residual oil distribution, achieved a synergistic effect of dominant channel control and deep mobilization in low-permeability zones during the supplemental water flooding stage, demonstrating stronger applicability and development performance.

**Figure 19:**
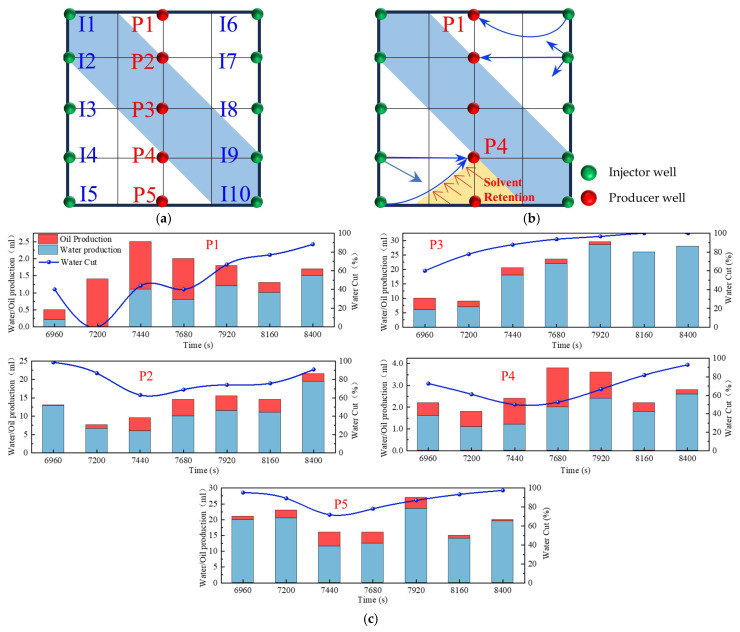
Schematic of single-well production performance variations after infill well pattern adjustment. (**a**) Well pattern deployment after infill adjustment. (**b**) Schematic of chemical slug retention in low-permeability zones. (**c**) Single-well production performance curves.

Based on the infill well pattern deployment (150 × 75 m) shown in Figure 19a and the production performance curves in Figure 19c, it was observed that the residual oil after chemical flooding was mainly enriched in low-permeability zones around the production wells. The streamline reversal caused by well pattern adjustment significantly improved the mobilization of the residual oil in low-permeability regions. The production performance showed three typical response types: (1) The P1, P2, and P5 well group exhibited significant increases in oil production (with P1 showing an increase of 46.7%), and the water cut showed a stage-wise reduction of 10–40%, indicating that streamline reorganization effectively mobilized the residual oil in low-permeability zones. (2) For well P5, due to the retention of the chemical slug (as shown in Figure 14b), the increase in oil production was limited (<25%), and water cut fluctuations were minimal, suggesting that reservoir heterogeneity reduced displacement efficiency. (3) For well P3, located in a high-permeability streak, the water cut rose to 100%, and oil production decreased by 40%, revealing a negative impact of the dominant channels. It is worth noting that, in the later stage of chemical flooding, the retained slugs near wells P4 and P5 were reactivated, resulting in sustained high oil production, although the water cut did not decrease significantly. This phenomenon confirms that the infill well pattern can continuously alter the pressure field distribution, gradually driving the retained chemical agents and improving the ultimate recovery of heterogeneous reservoirs.

#### 2.2.5. Variation Characteristics of Sweep Efficiency, Water Cut, and Oil Recovery Factor

During the water flooding stage, both experimental groups that directly implemented infill well pattern adjustment after water flooding experienced a water-free oil production period, followed by a rapid increase in the water cut. When the water cut reached approximately 90%, the recovery factors reached 32.32% (Scheme 1) and 33.51% (Scheme 2). At this point, the injected water preferentially flowed along the dominant channels in the high-permeability zones and rapidly broke through to the production wells, while large amounts of residual oil remained in the low-permeability zones, significantly limiting displacement efficiency.

After entering the infill chemical flooding stage, both systems effectively improved the fluid flow behavior and further mobilized the residual oil in unswept and weakly water-washed zones. In particular, the binary compound flooding scheme reduced the oil–water interfacial tension and optimized the mobility ratio, resulting in a decrease in the water cut from 90.53% to 51.34% and an increase in the recovery factor by 23.38%. However, due to the limited utilization of the slug, dominant flow channels were rapidly re-established during the subsequent water flooding stage, leading to a sharp rebound in the water cut. As a result, the displacement efficiency and mobilization performance remained insufficient.

In contrast, the heterogeneous compound flooding scheme exhibited a more favorable synergistic effect. After the injection of the HPC system, the water cut significantly decreased from 90.56% to 42.77%, and the recovery factor increased by approximately 29.95%. In this system, PPG particles effectively plugged the high-permeability channels, significantly suppressing water channeling and promoting the entry of the displacement fluid into medium- and low-permeability zones. As a result, a more balanced sweep was achieved, and the final recovery factor increased to 63.46%, demonstrating better recovery performance than the binary compound flooding scheme.

**Figure 20:**
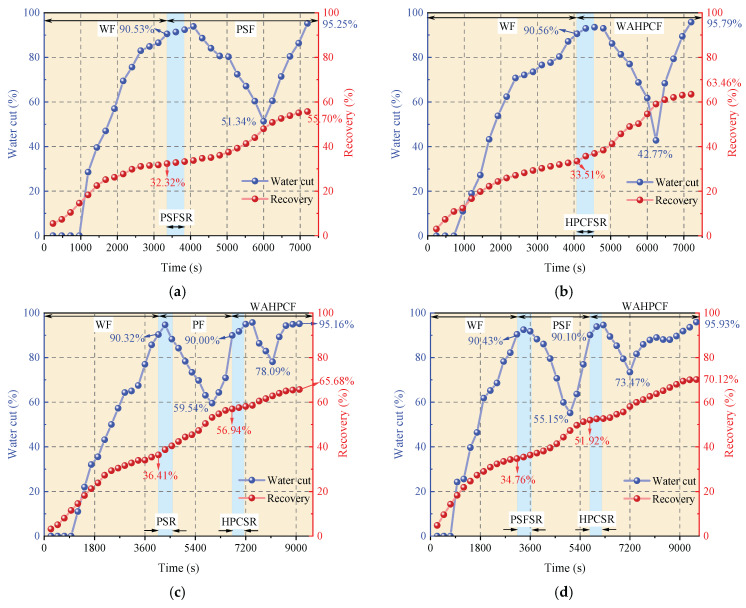
Water cut and recovery factor variation curves for the four infill chemical flooding experiments. (**a**) Scheme 1 (WF–WAPSF); (**b**) Scheme 2 (WF–WAHPCF); (**c**) Scheme 3 (WF–PF–WAHPCF); (**d**) Scheme 4 (WF–PSF–WAHPCF).

As shown in Figure 20b,c, the two schemes exhibited significant differences in water cut and recovery factor variations during the chemical flooding and infill heterogeneous flooding stages. In the chemical flooding stage, the water cut increased briefly at the beginning of slug injection and then decreased to 59.54% (Scheme 3) and 55.15% (Scheme 4), with the stage recovery factors increasing to 20.53% and 17.16%, respectively. The incremental recovery effects during this stage were generally comparable. As the subsequent water injection broke through the slug, the water cut increased again.

During the infill heterogeneous flooding stage, both schemes achieved further recovery enhancement. In Scheme 3, the water cut decreased to 78.09%, and the stage recovery factor increased to 65.68%; in Scheme 2, the water cut decreased to 73.47%, and the recovery factor rose to 70.12%. Scheme 4 demonstrated better performance in reducing the water cut and improving the recovery factor. The significant increase in recovery during this stage indicates that the binary flooding implemented in the early stage laid a solid foundation for subsequent infill well deployment and heterogeneous compound flooding. By improving the reservoir seepage field distribution, the sweep range was effectively expanded, and the mobilization of the residual oil in low-permeability zones was significantly enhanced.

**Figure 21:**
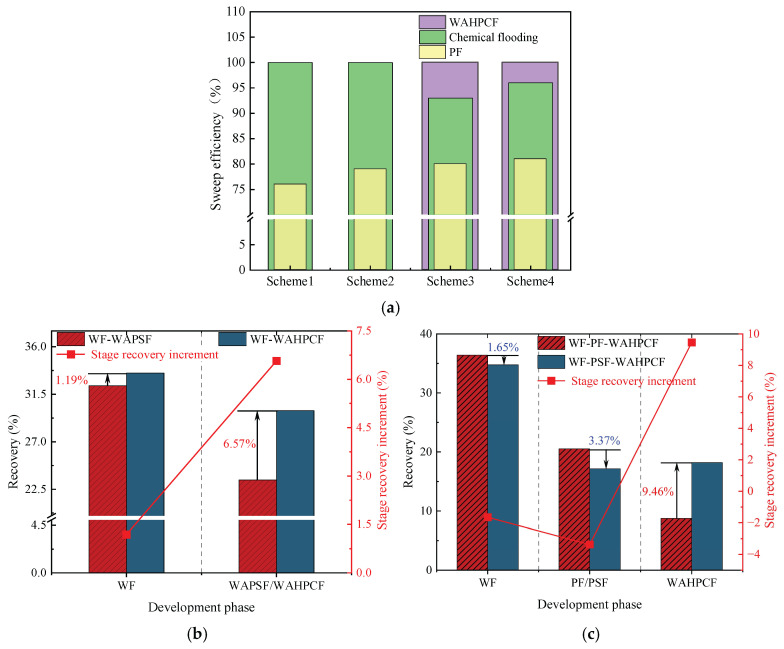
Sweep efficiency and recovery factor variations in the four infill chemical flooding experiments. (**a**) Sweep efficiency variation during the visualized experimental stage. (**b**) Stage recovery factor variation for Scheme 1 (WF-WAPSF) and Scheme 2 (WF-WAHPCF). (**c**) Stage recovery factor variation for Scheme 3 (WF-PF-WAHPCF) and Scheme 4 (WF-PSF-WAHPCF).

As shown in Figure 21b,c, two-dimensional visualized displacement experiments revealed significant differences in the evolution patterns of the water cut and recovery factor under different infill chemical flooding technologies. The results indicate that chemical flooding synergized with infill well patterns can effectively improve the development performance during the high-water-cut stage in the late period of water flooding, demonstrating a favorable synergistic displacement effect in reducing the water cut and enhancing recovery. During the direct infill chemical flooding stage, significant differences in displacement performance were observed among the different flooding agent systems. The heterogeneous system, with its high viscosity and flow diversion control capability, effectively plugged high-permeability channels, significantly weakened the water channeling phenomenon, and redirected the injected water into low-permeability zones, thereby expanding the sweep range of fluid flow. In comparison, although the binary compound flooding system exhibited certain interfacial regulation and emulsification capabilities, it was still limited in blocking high-permeability zones and supplementing displacement in weakly swept regions. Therefore, the heterogeneous flooding system achieved a more notable improvement in the recovery factor during this stage, with a more balanced and effective displacement process.

A further comparison reveals that, after infill well pattern adjustment, the stage recovery improvement of the direct infill schemes is superior to that of the schemes in which infill was implemented after chemical flooding. In particular, the direct infill heterogeneous flooding scheme demonstrates the best performance in terms of stage recovery enhancement, balanced fluid sweep, and the mobilization of the residual oil in weakly swept regions, owing to its stronger selective plugging capability in high-permeability zones and supplementary displacement efficiency in low-permeability zones. Although the direct infill heterogeneous flooding scheme has advantages in enhancing stage recovery, its final recovery factor is slightly lower than that of the synergistic scheme combining water flooding, chemical flooding, and infill heterogeneous compound flooding. This indicates that, although infill well pattern adjustment can improve displacement in weakly swept regions, its displacement depth and ultimate mobilization degree remain limited due to the absence of the effective optimization of the dominant flow channels and residual oil distribution patterns in the earlier chemical flooding stage.

In summary, the optimization of the displacement system is not only reflected in the improvement of the stage recovery factor but is also closely related to comprehensive factors such as the expansion of the sweep range, the optimization of the streamline distribution, and supplemental mobilization in weakly swept regions. Direct infill heterogeneous flooding demonstrates outstanding advantages in short-term recovery enhancement and displacement in weakly swept zones, whereas the water flooding–chemical flooding–infill heterogeneous flooding synergistic technique exhibits stronger overall advantages in optimizing the residual oil distribution and improving the total recovery, indicating its superior technical adaptability and application potential in the late stage of tertiary recovery in heterogeneous reservoirs.

### 2.3. Heterogeneous System Flooding Experiments with Various Infill Well Patterns

**Figure 22:**
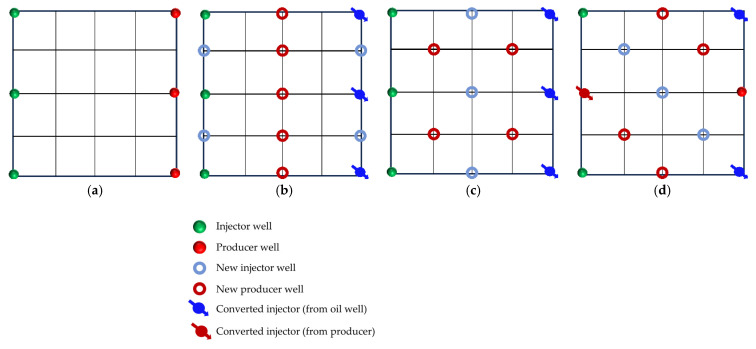
Infill well pattern variations in the three infill chemical flooding experiments. (**a**) Base well pattern: 300 × 150 m; (**b**) Scheme 1: linear row well pattern; (**c**) Scheme 2: inverted five-spot well pattern; (**d**) Scheme 3: staggered row well pattern.

Enhancing sweep efficiency and displacement efficiency is essential for improving the recovery factor of ultra-high-water-cut reservoirs in the post-tertiary recovery stage. For reservoirs with well-developed high-water-consuming zones, chemical flooding tends to balance seepage resistance, making it difficult to further expand sweep efficiency. This challenge can be addressed by infill well pattern adjustment to enhance the reservoir control capability. This study aims to investigate the influence of well pattern configurations on the performance of infill well pattern chemical flooding synergistic technologies. Based on a well pattern adjustment scheme for an ultra-high-water-cut reservoir in a block of Shengli Oilfield, three heterogeneous flooding infill well pattern configurations are designed: a linear pattern, an inverted five-spot pattern, and a staggered row pattern (Figure 22). This study focuses on the matching relationship between infill well patterns and high-permeability streaks, changes in dominant injection–production streamlines, and the single-well control capability after infill adjustment. It also analyzes the residual oil distribution characteristics, water cut, and recovery factor evolution after the implementation of infill chemical flooding synergistic development.

#### 2.3.1. Visual Experimental Results

All three infill well pattern configurations improved the fluid migration behavior in the reservoir to varying degrees. By shortening the injection–production well spacing and optimizing the injection–production relationship, the infill well patterns significantly enhanced the interference between the injection and production wells and increased the local displacement pressure gradient, thereby effectively increasing the streamline density and adjusting the flow direction. This adjustment not only expanded the sweep range of the injected water and the chemical slug, improving the uneven advancement dominated by the dominant channels in the late stage of tertiary recovery, but also further strengthened the supplemental mobilization of the residual oil in weakly swept zones. Meanwhile, the control space formed by the infill well pattern optimized the distribution of the displacement fluids, enabling the chemical slug to more effectively act on residual oil-enriched regions. The sweep efficiency and utilization of the slug significantly improved, reducing slug escape and rapid breakthrough through high-permeability channels, as well as allowing for the full realization of the combined displacement effect of the slug and subsequent water injection.

**Figure 23:**
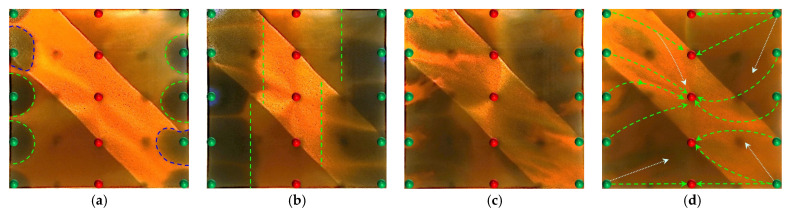
Scheme 1 in the infill heterogeneous compound flooding experiment with well pattern adjustment. (**a**) Initial stage of HPC injection. (**b**) Final stage of HPC injection. (**c**) Initial stage of subsequent water injection. (**d**) The 95% water cut stage.

**Figure 24:**
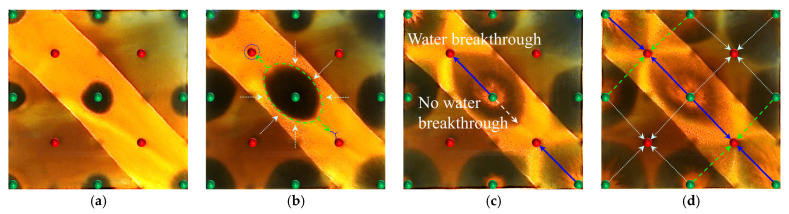
Scheme 2 in the infill heterogeneous compound flooding experiment with well pattern adjustment. (**a**) Initial stage of HPC injection. (**b**) Final stage of HPC injection. (**c**) Initial stage of subsequent water injection. (**d**) The 95% water cut stage.

In terms of specific well pattern comparisons, the different schemes exhibited distinct characteristics in the streamline distribution and displacement behavior (Figure 23 and Figure 24). In the linear row well pattern, the dominant injection–production streamlines were concentrated, with some reversal observed. The high-permeability streaks had a significant blocking effect on fluid flow, leading to a sharp increase in the water cut of the production wells after water breakthrough. The single-well control capability was limited, and displacement efficiency was relatively low. The inverted five-spot well pattern showed a divergent streamline distribution, which was favorable for improving overall reservoir utilization. However, the shorter spacing between the injection and production wells allowed the chemical slug to more easily break through the high-permeability streaks, accelerating water channeling during the subsequent water flooding stage. As a result, the sweep effectiveness of the slug was limited, its utilization was relatively low, and slug retention occurred.

**Figure 25:**
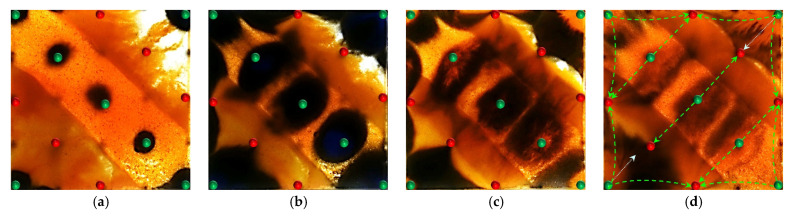
Scheme 3 in the infill heterogeneous compound flooding experiment with well pattern adjustment. (**a**) Initial stage of HPC injection. (**b**) Final stage of HPC injection. (**c**) Initial stage of subsequent water injection. (**d**) The 95% water cut stage.

In contrast, the staggered row well pattern scheme exhibited the best overall performance in terms of fluid regulation and displacement efficiency (Figure 25). The turning angles of the main injection–production streamlines were maintained between 45° and 90°, effectively weakening the interception effect of high-permeability streaks and slowing the formation of dominant seepage channels. Meanwhile, under this scheme, the chemical slug was more uniformly distributed, with higher utilization and less retention. The streamline distribution was dense and balanced, significantly improving the sweep uniformity during the displacement process. Especially in the late stage of tertiary recovery, the staggered row well pattern markedly expanded the sweep area in non-dominant zones and promoted the efficient mobilization of the residual oil, resulting in a continuous improvement in the recovery factor. This scheme, through streamline reorientation and flow diversion, enabled the chemical slug to effectively enter the originally weakly swept regions, achieving a synergistic effect of “optimized injection–production relationship—expanded sweep area—enhanced slug effectiveness.”

#### 2.3.2. Variation Characteristics of Sweep Efficiency, Water Cut, and Oil Recovery Factor

**Figure 26:**
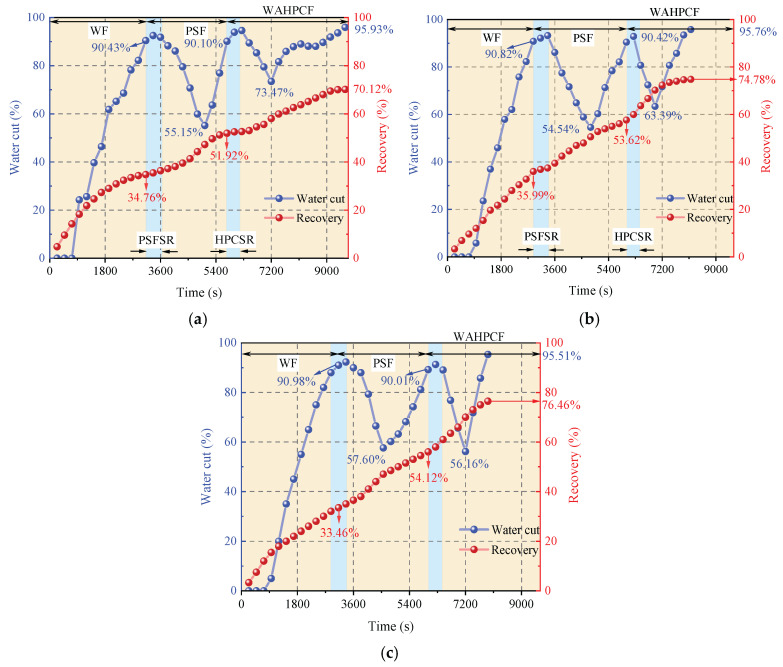
Water cut and oil recovery variations in the three sets of well pattern infill chemical flooding experiments. (**a**) Scheme 1: direct line-drive well pattern. (**b**) Scheme 2: inverted five-spot well pattern. (**c**) Scheme 3: staggered line-drive well pattern.

**Figure 27:**
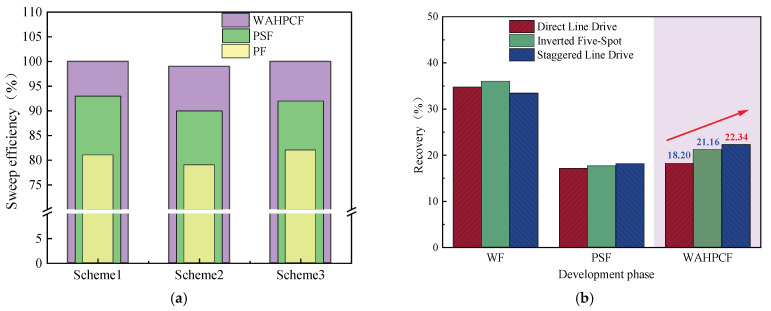
Variations in sweep efficiency and oil recovery in three chemical flooding experiments with different well pattern infill arrangements. (**a**) Sweep efficiency variation. (**b**) Incremental oil recovery during the stage.

During the infill well pattern chemical flooding stage, all three schemes achieved significant reductions in the water cut and further improvements in the recovery factor, although differences in displacement performance were observed (Figure 26 and Figure 27). In Scheme 1, the water cut decreased from 90.10% at the end of the chemical flooding stage to 73.47%, a reduction of 16.63%. Meanwhile, the recovery factor increased from 51.92% to 70.12%, with a stage recovery increase of 18.20%. The decrease in the water cut and the increase in the recovery factor indicate that the infill well pattern and heterogeneous flooding system partially improved the fluid flow behavior, effectively blocked the dominant channels, and initiated the mobilization of the residual oil in low-permeability zones. However, the relatively limited increase in recovery during this stage suggests that there is still room for improving slug utilization and the mobilization degree in low-permeability areas.

In Scheme 2, the water cut rapidly decreased from 90.42% at the end of chemical flooding to 63.39%, representing a reduction of 27.03%. The oil recovery increased from 53.62% to 74.78%, with an incremental recovery of 21.16%, which is significantly higher than that of Scheme 1. The substantial decrease in the water cut and the increase in oil recovery reflect a good synergistic effect between the heterogeneous composite flooding system and the infill well pattern. This effectively blocked the high-permeability dominant channels and enhanced the displacement capacity for the remaining oil in low-permeability zones, leading to a more balanced displacement process.

In comparison, Scheme 3 exhibited a more significant oil displacement effect during this stage. The water cut rapidly decreased from 90.01% at the end of chemical flooding to 56.16%, with a reduction of 33.85%. The oil recovery increased from 54.12% to 76.46%, resulting in an incremental recovery of 22.34%, which was significantly higher than that of Scheme 1 and Scheme 2. The results indicate that the staggered well pattern arranged parallel to the high-permeability zone can effectively enhance the oil recovery in high-water-cut reservoirs.

## 3. Conclusions

Based on two-dimensional visual physical simulation experiments, this study systematically evaluated the oil displacement performance and mechanisms of the optimized and synergistic application of infill well pattern and heterogeneous composite flooding technologies in response to challenges such as intensified reservoir heterogeneity, inefficient displacement dominated by preferential flow channels, and a dispersed distribution of the remaining oil after tertiary recovery in mature oilfields. The main conclusions were obtained:

1. When the effectiveness of a single chemical flooding technique is limited, the optimized combination of infill well pattern and heterogeneous composite flooding can effectively improve the unbalanced displacement pattern dominated by preferential flow channels in the late stage of water flooding. The results indicate that the synergistic approach outperforms traditional single chemical flooding strategies in enhancing sweep efficiency and mobilizing the remaining oil in poorly swept zones, providing an effective technical path for improving oil recovery in the high-water-cut stages of complex heterogeneous reservoirs.
2. The heterogeneous composite flooding system (HPC) exhibits a strong synergistic effect of plugging and displacement under infill well pattern conditions. Experimental results show that the HPC slug can effectively suppress breakthrough in high-permeability channels and promote the diversion of the displacement fluid toward medium- and low-permeability zones, thereby significantly enhancing the sweep volume and the mobilization efficiency of the remaining oil.
3. Significant differences exist in the synergistic oil displacement performance among different well pattern configurations. Among them, the combination of the staggered line-drive well pattern and the HPC system achieves the best results, with an incremental oil recovery of 22.34% and a water cut reduction of 33.85%. This configuration demonstrates prominent advantages in displacement uniformity and overall recovery enhancement. The staggered line-drive well pattern optimizes the fluid streamline distribution, effectively weakens the conductivity of preferential flow channels, improves slug utilization, and enhances displacement efficiency in poorly swept zones. It outperforms the other two infill well pattern schemes, highlighting the technical significance of rational well pattern design in combination with heterogeneous flooding for enhanced oil recovery.
4. The displacement condition during early-stage chemical flooding has a significant impact on the subsequent mobilization performance of heterogeneous composite flooding under the infill well pattern. Compared with the infill scheme following polymer flooding, the scheme following binary composite flooding demonstrates better supplementary displacement performance in low-permeability zones due to the sustained action of the surfactant, resulting in a greater improvement in the final oil recovery. This reflects the amplifying and inheriting relationship between the infill well pattern and chemical flooding technologies in enhancing development performance.

## 4. Materials and Methods

### 4.1. Fundamentals of Experimental Design

A physical simulation is an important approach for studying reservoir development mechanisms, and its theoretical basis lies in similarity theory. By using dimensionless parameters, the physical phenomena between the experimental model and the actual reservoir are correlated to ensure consistency in terms of geometric, kinematic, and dynamic similarities. Since it is difficult to account for all similarity criteria during model construction and experimental processes, it is necessary to select the similarity numbers that have a major influence on the simulation results, while less critical parameters can be reasonably neglected [27].

Based on reservoir engineering similarity theory, this study established a systematic set of similarity criteria for two-dimensional physical simulation experiments. By coordinating multiple parameters across three dimensions—geometric, dynamic, and kinematic similarities—a cross-scale similarity from microscopic seepage to macroscopic reservoir development was achieved, ensuring that the physical model accurately reflected the dynamic characteristics of actual reservoir development. During model construction, key parameters such as the model dimensions, crude oil viscosity, injection rate, and well spacing were adjusted to scale up or down the production characteristics of the actual reservoir while ensuring that these parameters met the similarity criteria. On the basis of independently analyzing existing similarity numbers, the following parameters were considered in detail [48,50,51]:

1. Geometric similarity

Geometric similarity ensures that the spatial scale of the experimental model matches the physical characteristics of the actual reservoir. The specific geometric similarity criteria are as follows:

(1) $$\gamma \left(\frac{L_{m}}{L_{r}}\right)=1$$

where *L_m_* and *L_r_* represent the dimensions of the model and the actual reservoir, respectively. This formula ensures that the size ratio between the model and the reservoir is consistent, thereby guaranteeing similarity in the well pattern configuration and sand body distribution characteristics.

2. Production pressure differential similarity

The production pressure differential plays a critical role in fluid flow and recovery efficiency. To ensure consistency between the model and the actual reservoir in terms of the production pressure differential, the following similarity control formula is applied:

(2) $$\gamma \left(K_{a}\Delta p/\eta \mu L\right)=1$$

In addition, the relationship between the pressure differential and other physical properties (such as viscosity, permeability, and porosity) can be further expressed as

(3) $$\gamma \left(\Delta p\right)=\gamma \left(\mu \right)\gamma \left(\eta \right)\gamma \left(L\right)/\gamma \left(K_{a}\right)=\gamma \left(\Delta \rho \right)\gamma \left(L\right)$$

where *μ* is the fluid viscosity; *η* is the dynamic viscosity; *L* is the characteristic length; *K_a_* is the permeability; ∆*p* is the production pressure differential; and *ρ* is the fluid density. By adjusting the relevant parameters, the model can maintain consistency with the actual reservoir in terms of the production pressure differential.

3. Injection volume similarity

The injection volume is a critical factor affecting fluid injection and production behavior during reservoir development. To ensure similarity between the model and the actual reservoir in terms of the injection volume, the following dimensionless injection control formula is applied:

(4) $$\gamma \left(\frac{I_{i}}{\eta L^{2}}\right)=1$$

In addition, the injection volume is also related to fluid properties such as viscosity and permeability, as well as the pressure differential. The detailed control formula is given as follows:

(5) $$\gamma \left(I_{i}\right)=\gamma \left(L^{2}\right)\gamma \left(\eta \right)=\gamma \left(L^{2}\right)\gamma \left(K_{a}\right)\gamma \left(\Delta \rho \right)/\gamma \left(\mu \right)$$

where *I_i_* is the injection volume. By dynamically adjusting the injection rate and volume through real-time monitoring, injection volume similarity can be ensured.

By converting the actual reservoir parameters into dimensionless forms and substituting them into the mathematical model, the relevant parameters for the physical simulation experiment were calculated. Meanwhile, due to limitations in experimental materials, equipment, and environmental conditions, it was not possible to satisfy all similarity criteria simultaneously. Therefore, certain parameters were reasonably adjusted based on the similarity principles and experimental conditions. A comparison between the key experimental parameters and actual reservoir parameters is shown in Table 4.

### 4.2. Materials

Simulated water: Both the saturated water and the displacement water used in the experiment were ultrapure water.

Simulated oil: The simulated oil was prepared by mixing industrial lubricating oil and kerosene. Its viscosity was 45 mPa·s at 26 °C, and the density was 0.83 g/cm^3^.

Polymer: The polymer used in the experiment was provided by Shengli Oilfield. It was partially hydrolyzed polyacrylamide (HPAM), with a molecular weight of 2.0 × 10^7^ and a solid content of 89.55%. The polymer solution was prepared with ultrapure water at a concentration of 1000 mg/L.

Surfactant: The surfactant used in the experiment was petroleum sulfonate (molecular formula: C_23_H_38_SO_3_M), also provided by Shengli Oilfield.

B-PPG (bulk preformed particle gel): The B-PPG was supplied by Shengli Oilfield. It had an elastic modulus of 10.3 Pa and a particle size of 100–150 mesh. The B-PPG was added to the simulated water in dry powder form and formed a gel through water absorption and swelling to improve displacement performance.

Glass beads: The glass beads were made of borosilicate glass with a mesh size of 60–100 and an average particle diameter of 150 μm.

Dyes: Two types of dyes were used in the experiment, namely, methyl orange (molecular formula: C_14_H_14_N_3_NaO_3_S) and methylene blue (molecular formula: C_37_H_27_N_3_Na_2_O_9_S_3_), which were used to stain the displacement water and the HPCF system, respectively.

**Figure 28:**
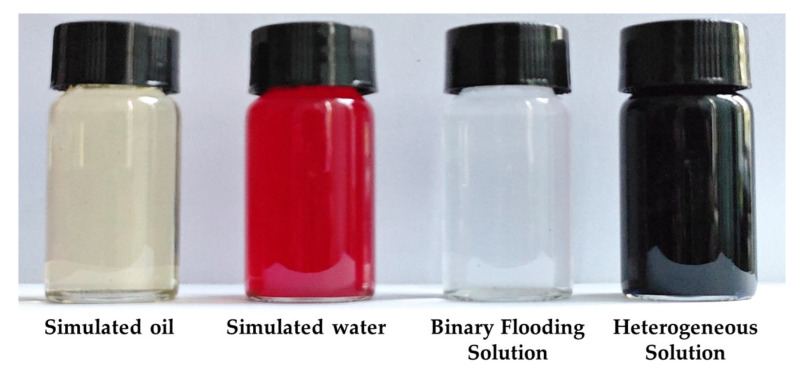
Displacement solutions used in the visualization experiment (simulated oil, simulated water, binary composite flooding solution, and heterogeneous solution).

In this study, the formulation of chemical flooding agents was designed based on field-proven parameters widely adopted in Shengli Oilfield and by referring to the research findings of Zhang et al. [52] and Wu et al. [61], which focus on enhancing oil recovery during the high-water-cut stage. This ensured that the proposed flooding systems maintained strong field applicability under laboratory conditions. Considering the development characteristics of point bar deposits and the distribution heterogeneity of the reservoir architecture in Shengli Oilfield, three typical types of chemical flooding systems were designed: polymer flooding, binary flooding, and heterogeneous phase composite flooding. The specific compositions and concentrations are listed in Table 5. The polymer flooding system consisted of partially hydrolyzed polyacrylamide (HPAM) at a concentration of 1000 mg/L. On this basis, the binary system incorporated an anionic surfactant ST1-101 at a concentration of 1000 mg/L, aiming to reduce oil–water interfacial tension and enhance emulsification and displacement efficiency. Furthermore, the heterogeneous phase composite flooding system introduced 500 mg/L of the branched preformed particle gel (B-PPG), which regulated the streamline distribution through the dynamic plugging effect of the gel particles, as shown in Figure 28.

### 4.3. Experimental Setup

**Figure 29:**
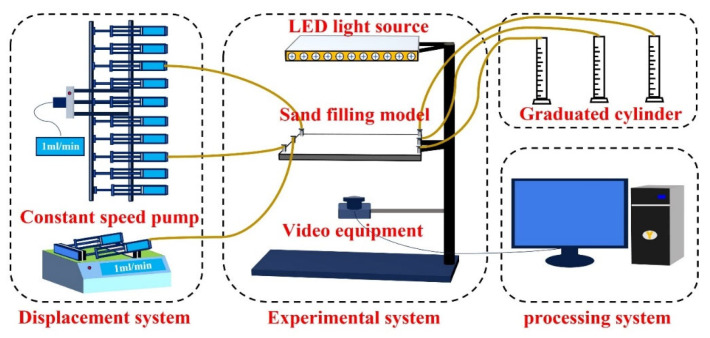
Schematic diagram of the experimental setup.

The experimental setup is shown in Figure 29. The entire apparatus is divided into three systems: the experimental system, the displacement system, and the processing system. The experimental system includes a two-dimensional sand-packed model, an experimental operation table, a high-brightness LED light source, and a high-resolution camera. The displacement system consists of one 10-channel constant-rate injection pump and two dual-channel constant-rate injection pumps. The injection pumps have a flow rate range of 0.1–25.00 mL/min, a working pressure range of 0–3 MPa, and a precision of ±0.05%. These pumps are connected to the experimental system via pipelines and switching valves. The processing system includes a measuring cylinder and a computer-based acquisition and processing system, which is used to record experimental data and generate images in real time.

**Figure 30:**
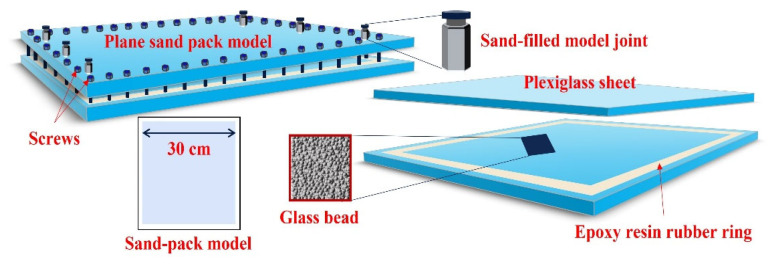
Schematic diagram of the two-dimensional sand-filled model.

A custom-built two-dimensional sand-packed model is constructed, as shown in Figure 30. The model is composed of two 5 cm thick plexiglass plates and a rubber sealing ring. The plexiglass provides excellent transparency, allowing for a clear observation of fluid movement and displacement fronts during the experiment. A 5 mm deep groove is etched into the bottom plexiglass plate to accommodate a rubber sealing ring made of a 3 cm wide high-pressure- and corrosion-resistant silicone material. The upper and lower plexiglass plates are fastened together using screws, compressing the rubber ring to achieve effective sealing. The working pressure range of the model is 0-1.5 MPa. The overall dimensions are 35 cm × 35 cm, with an actual sand-packing area of 30 cm × 30 cm and a packing thickness of 8 mm. The model is equipped with 25 prefabricated well ports, enabling the flexible deployment of various well pattern configurations.

### 4.4. Experimental Procedures

**Figure 31:**
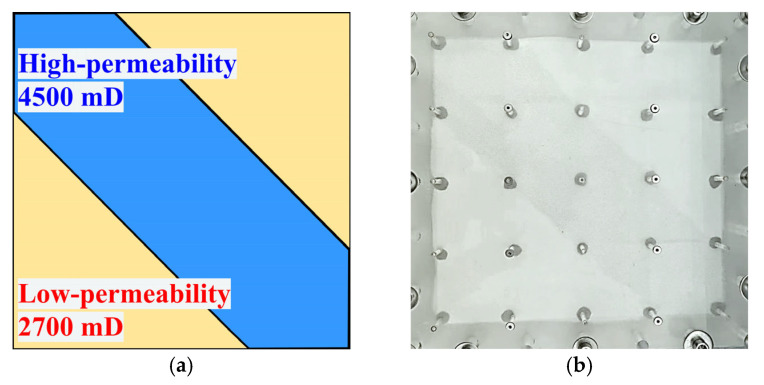
(**a**) Distribution of high- and low-permeability zones in the experimental model. (**b**) Plane sand-packed model.

In the experiment, the point bar sedimentary structure was characterized by the alternating distribution of high-permeability streaks and low-permeability zones, a type of heterogeneity that significantly influences fluid flow behavior and reservoir performance. Based on the sedimentary features of a point bar developed in a specific block of Shengli Oilfield, a heterogeneous physical model with distinct high-permeability streaks was constructed to replicate the representative characteristics of such reservoirs, as shown in Figure 31. The low-permeability zones were packed with 100-mesh glass beads, resulting in a permeability of approximately 2700 mD, while the high-permeability zones were packed with 60-mesh glass beads, yielding a permeability of about 4500 mD. Throughout the experiment, oil and liquid production volumes were recorded every 240 s, and the displacement front and streamline evolution were continuously monitored in real time.

The detailed experimental procedure is as follows:

1. Clean the glass beads using ultrapure water to remove impurities, and then dry them at a high temperature (120 °C) to ensure consistent wettability. The glass beads of different mesh sizes used in the experiment are shown in Figure 30.
2. Weigh the cleaned glass beads to ensure the same sand-packing mass across all experimental groups. Pack the beads into the model zone by zone, ensuring similar porosity among the experiments. After repeated vibration and compaction, place the upper glass plate, tighten the screws, and further compress and seal the glass beads.
3. Use a vacuum pump to evacuate air from the sand-packed model, ensuring a vacuum state and checking the airtightness of the system.
4. Inject ultrapure water into the sand-packed model using a multi-cylinder constant-rate injection pump at a steady rate of 3 mL/min until the model is fully saturated. Calculate the porosity using the material balance method. Afterward, leave the model undisturbed for 4 h to stabilize the simulated formation water.
5. Inject the simulated oil at a constant rate of 2 mL/min until 100% oil is produced at the model outlet. Record the injected oil volume as the geological reserves. Let the model stand for 24 h to balance the fluid distribution.

The physical properties of the model after the pre-experiment preparation are listed in Table 6.

### 4.5. Experimental Data Processing

1. Method Development

This study innovatively integrates image segmentation techniques with clustering algorithms to establish an intelligent analysis method applicable to two-dimensional displacement experiments. Based on experimental images, the method constructs a quantitative characterization system of the displacement agent sweep area and remaining oil distribution through steps such as image preprocessing, color space transformation, boundary enhancement, and clustering segmentation. By calculating the pixel proportions of different image regions, the dynamic variation characteristics of various zones during the displacement process are extracted. This method overcomes the technical limitations of traditional approaches that rely on manual identification and binary processing, significantly improving analysis efficiency and accuracy.

2. Image Processing Workflow

**Figure 32:**
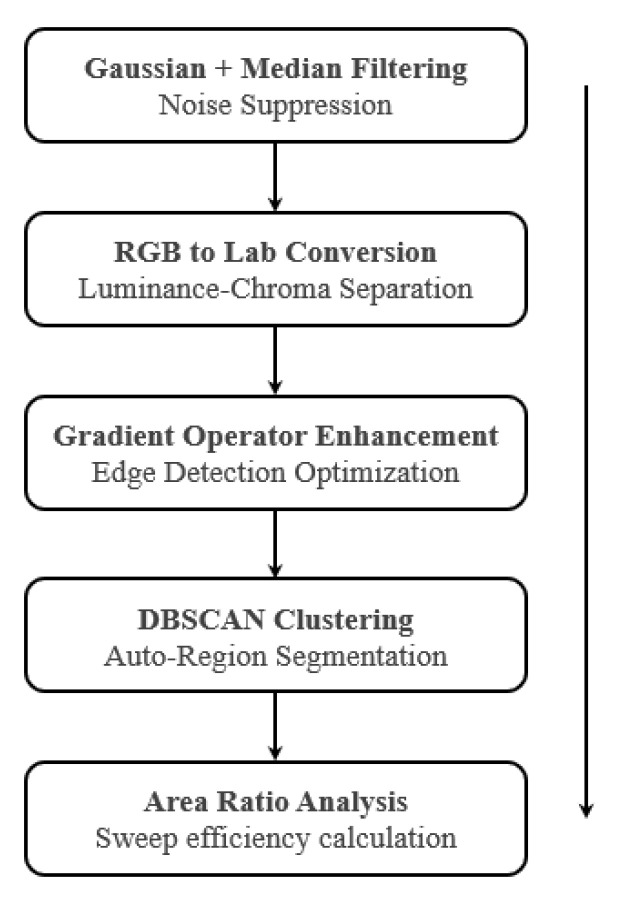
Image processing procedure.

A multi-step image processing approach was employed in this study for a systematic analysis of experimental images (Figure 32). First, Gaussian filtering combined with median filtering was used to suppress image noise, effectively reducing the interference caused by imaging devices and environmental conditions. Then, the image was transformed from RGB to Lab color space, utilizing the separation of the luminance and chromaticity channels to address uneven lighting. Subsequently, gradient operators (Sobel/Laplacian) were applied for edge enhancement to highlight key features such as the displacement front and oil–water interface. Based on this, the DBSCAN clustering algorithm was used for automatic image segmentation, which demonstrates good adaptability to irregular shapes and blurred boundaries. Finally, using the segmentation results and a standardized color feature library, a quantitative analysis of the distribution of displacement and residual oil regions was achieved through pixel area ratio calculation. This approach provided a reliable basis for studying the dynamic distribution of the remaining oil and significantly enhanced the accuracy of displacement front identification and the robustness of the analytical results.

3. Validation of Effectiveness

**Figure 33:**
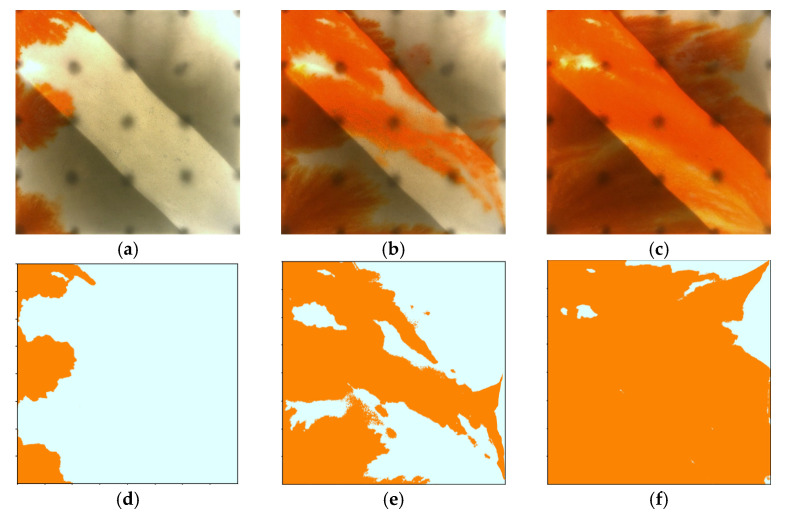
Experimental image processing technique. (**a**–**c**) Original images. (**d**–**f**) Image segmentation using the DBSCAN clustering algorithm.

As shown in Figure 33, by processing and analyzing experimental images at different displacement front positions, the dynamic evolution characteristics of the residual oil distribution during the displacement process were successfully extracted. The experimental results indicate that, compared with traditional binarization methods, the proposed method controls the calculation error within 3%. The DBSCAN clustering algorithm demonstrates strong robustness when dealing with complex conditions such as uneven lighting and blurred boundaries. Furthermore, the standardized analysis method based on the color feature library is applicable to video image processing.

Through macroscopic visual physical simulation experiments, this method enables image acquisition and performance evaluation of the displacement process, establishing a technical framework that transitions from a qualitative observation to a quantitative analysis. It provides a novel research tool for evaluating the effectiveness of chemical flooding.

## Figures and Tables

**Table 1:** Comparison of zero-shear viscosity, infinite-shear viscosity, and steady-state viscosity of polymer, binary composite, and heterogeneous composite displacement systems.

System	Zero-Shear Viscosity (mPa·s)	Infinite-Shear Viscosity (mPa·s)	Viscosity mPa·s
Polymer	2752.69	31.83	25.85
Binary Composite Flooding	1859.88	33.37	24.55
Heterogeneous Composite Flooding	6299.85	41.56	52.03

**Table 2:** Sweep efficiency and oil recovery factor under different pore volumes injected during the water flooding stage.

Stage	0.1 PV	0.3 PV	0.6 PV	0.8 PV
Sweep Efficiency (%)	17.56	47.61	68.42	87.15
Recovery Factor (%)	7.23	19.54	26.81	32.32

**Table 3:** Sweep efficiency and recovery factor at different stages of chemical flooding.

Displacement Process	Stage	Initial Stage of Slug Injection	End of Slug Injection	Initial Stage of Subsequent Water Injection	Water Cut up to 95%
WF-WAPSF	Sweep Efficiency	72.56%	89.63%	93.21%	95.72%
	Recovery factor	34.17%	38.61%	47.56%	55.70%
WF-WAHPCF	Sweep Efficiency	74.24%	92.15%	100%	—
	Recovery factor	33.51%	42.38%	56.14%	63.46%

**Table 4:** Comparison of experimental parameters with actual reservoir parameters.

Parameter	Unit	Reservoir Data	Simulation Data
Temperature	°C	80	26
Pressure	MPa	12.56	0.1
Porosity	%	31	30
Permeability	10^−3^ μm	3650	3500
Oil Density	g/cm^3^	0.89	0.86
Oil Viscosity	mPa∙s	44.6	45
Water Density	g/cm^3^	1.02	1.02
Water Viscosity	mPa∙s	0.46	0.46
Injection Rate	(m^3^/d) or (mL/min)	160	1.5

**Table 5:** Chemical agent formulation.

Solution Name	Composition	Concentration (mg/L)	Solution Color
Polymer Solution	HPAM	1000	Colorless and transparent
Binary Flooding Solution	HPAM	1000	Colorless/blue
	Surfactant	1000	
Heterogeneous Solution	HPAM	1000	Blue
	Surfactant	1000	
	B-PPG	500	

**Table 6:** Basic parameters of the two-dimensional sand-packed model experiment.

	Chemical Flooding Method	Infill Well Pattern	Pore Volume (mL)	Porosity	Reserves (mL)	Injection Rate
1	WF-WAPSF	Aligned row pattern	52.0	0.330	264	1.5 mL/min
	WF-WAHPCF		52.0	0.325	262	
	WF-PF-WAHPCF		53.0	0.340	265	
	WF-PSF-WAHPCF		49.0	0.290	258	
2	WF-PSF-WAHPCF	Aligned row pattern	50.5	0.314	261	
	WF-PSF-WAHPCF	Reverse five-spot pattern	52.5	0.338	263	
	WF-PSF-WAHPCF	Staggered row pattern	51.0	0.319	261	

## Data Availability

The data of this article are available from the corresponding author upon reasonable request.

## Abbreviations

The following abbreviations are used in this manuscript:

WF	Water flooding
PF	Polymer flooding
PS	Polymer–surfactant binary composite system
PSF	Polymer–surfactant flooding
WAPSF	Well pattern densification and adjustment combined with PS flooding
HPC	Heterogeneous phase composite system
HPCF	Heterogeneous phase composite flooding
WAHPCF	Well pattern densification and adjustment combined with HPC flooding
